# The evolution of hereditary cancer genetic counselling: mainstreaming, service redesign and patient experience in Lynch syndrome

**DOI:** 10.1007/s10689-026-00597-6

**Published:** 2026-08-29

**Authors:** Kelly Kohut, Sarah Cable, Alekhya Ashokan, Stefania Vicari, Hannah Ditchfield, Julie Young, Ben Bux, Fiona Lalloo

**Affiliations:** 1 Department of Clinical and Biomedical Sciences, University of Exeter Medical School, Exeter, UK; 2 Centre for Genomic Medicine, St. George’s University Hospitals NHS Foundation Trust, London, UK; 3 Gastroenterology, St. George’s University Hospitals NHS Foundation Trust, London, UK; 4 Department of Communication and Media, Loughborough University, Loughborough, UK; 5 Patient Representative, London, UK; 6 Manchester Centre for Genomic Medicine, St Mary’s Hospital Manchester, Manchester, UK

**Keywords:** Genetic counselling, Mainstreaming, Genetic testing, Shared decision-making, Lynch syndrome, Patient and public involvement (PPI)

## Abstract

Genetic counselling for hereditary cancer predisposition has evolved substantially over more than three decades, driven by advances in genomic technologies and the growing use of tumour and germline testing. These developments have expanded access to hereditary cancer testing, treatment and prevention, while also introducing new clinical, psychosocial and system-level challenges. This review examines the transition from traditional specialist-led genetic counselling to mainstreamed testing pathways. Using Lynch syndrome as a paradigm, we explore how service redesign, personalised risk communication and patient experience intersect in contemporary hereditary cancer care. This invited review synthesises clinical, academic and patient perspectives on the evolution of hereditary cancer genetic counselling. Lived experiences of Lynch syndrome were incorporated through patient and public involvement contributors involved in manuscript development and interpretation. Mainstreamed testing has expanded access to genomic information and strengthened opportunities for cascade testing and prevention within families. However, it has also increased testing volumes, complexity of results interpretation and reliance on non-genetics clinicians. As a result, effective care requires close coordination between genetics and cancer services, alongside support for shared decision-making, family communication and psychosocial needs. This review uniquely integrates clinical evidence and lived experience to highlight how mainstreaming has transformed the delivery of hereditary cancer genetic counselling but not the need for specialist genetics expertise. Lynch syndrome illustrates how genomics can be integrated into routine cancer care while maintaining personalised counselling and family-centred follow-up. These principles are relevant across hereditary cancer predispositions and inform the future development of genetic counselling services.

**Supplementary Information:**

The online version contains supplementary material available at https://doi.org/10.1007/s10689-026-00597-6.

## Introduction

Genetic counselling for hereditary cancer predispositions has been established for more than 35 years, first emerging in the UK, North America, Australia, the Netherlands and Nordic countries from the late 1980s [1–3]. Rapid expansion followed in the mid-1990s with the discovery of *BRCA1* [4]*, BRCA2* [5] and the mismatch repair genes associated with Lynch syndrome [6–10]. Since then, practice has evolved in successive waves, driven by advances in genomic medicine and oncology [11–13]. Models of service delivery and professional roles have varied internationally and provision remains uneven, with some countries still lacking formal training programmes and clinical services [14]. Even in settings with established services, demand has increasingly outpaced capacity [15, 16]. 

The therapeutic relevance of genomic testing has transformed this into a routine test at the time of diagnosis for many tumour types [17–22]. Tumour profiling can influence treatment choice, prognosis, long-term planning and the identification of relatives with inherited risk [23–25]. In combination with lower costs advances in laboratory methods and increasing public awareness, this has contributed to a shift towards mainstreamed genomic testing. Mainstreaming refers to the integration of genomic testing into routine cancer care pathways, whereby non-genetics clinicians undertake pre-test discussions and initiate testing, with specialist genetics services providing support for complex cases and post-test management [16, 26–29].

The aim of this review is to examine the evolution from traditional hereditary cancer genetic counselling to mainstream integration within routine cancer care pathways, using Lynch syndrome as a paradigm. We focus on how service redesign, personalised risk communication and patient experience have shaped contemporary hereditary cancer care and we consider the implications for scalable and equitable implementation of mainstream testing in coordination with specialist genetics services.

### Traditional model of genetic counselling

Traditionally, hereditary cancer genetic counselling was delivered in specialist clinical settings by consultant geneticists and genetic counsellors working closely with molecular geneticists, family history nurses, specialist nurses and clinical psychologists. Referrals were largely restricted to individuals with a strong personal or family history of cancer.

Counselling in this model was based on limited knowledge of hereditary cancer risks at the time, when testing was typically limited to single genes using approaches such as Sanger sequencing. As a result, counselling was often relatively standardised, with limited differentiation between genes or between families with different risk profiles. In Lynch syndrome, for example, individuals were historically managed using broadly similar recommendations before gene-specific management guidance was developed.

Risk management in the traditional model centred on surveillance with techniques such colonoscopy and mammography aimed at earlier detection [30–32] or surgical removal of at-risk organs aimed at cancer risk reduction [33, 34]. Although effective, surgical risk-reduction strategies may have important long-term consequences. In Lynch syndrome, these can include altered bowel function following colectomy or early menopause following gynaecological surgery, which can be clinically significant despite partial mitigation with hormone replacement therapy [35].

Appointments were usually conducted in person and involved detailed pre-test genetic counselling, including elicitation of personal and family histories to assess the likelihood of inherited cancer susceptibility. The benefits and limitations of testing for the individual were discussed, along with the implications of sharing results with relatives. Informed consent was typically obtained for a specific diagnostic or predictive test using a signed, paper form.

This model was time-intensive [1]. Two appointments, often lasting 45–60 minutes each, were generally required to support informed consent and results interpretation. Even then, the model assumed that individuals could process complex information in a clinical encounter and that they had adequate health literacy, emotional readiness and stable family relationships [1]. These assumptions were later recognised as frequently unmet [36]. The amount of information provided could be overwhelming, particularly when counselling followed a new cancer diagnosis [37, 38]. Recognition of these limitations contributed to the development of more flexible service models, including mainstream pathways that combine shorter pre-test discussions with patient-facing resources with referral to specialist genetics services for focussed, personalised discussion about results implications.

### Advanced counselling skills

International genetic counselling training programmes have consistently emphasised the integration of health psychology and counselling theory to support individuals and families facing hereditary cancer risk [2]. These frameworks recognise the multidimensional psychological challenges of genetic testing, including fear, cancer-related anxiety, adjustment to a genetic diagnosis and guilt related to the potential transmission of inherited risk to offspring [2, 39–41]. For many individuals, these concerns extend beyond immediate clinical decisions to broader questions about future cancer risk, uncertainty and coping style [42–46].

Although people are generally resilient, individuals with a high-risk cancer predisposition may experience complex psychological adjustment [47]. The need to minimise decision regret, especially when considering optional and potentially irreversible interventions such as risk-reducing surgery where there may be no single 'right’ choice or optimal timing, has contributed to the development of more psychologically informed approaches, sometimes described as psychotherapeutic genetic counselling [48]. These approaches place greater emphasis on emotional processing, values clarification and longitudinal support.

Evidence from decision science research further indicates that even when individuals understand statistical risk information, decisions are shaped by emotional responses, heuristics, the views of trusted others and perceived gains and losses associated with risk [48–53]. Recognising and addressing these influences is therefore central to effective genetic counselling and shared decision-making in hereditary cancer care.

Given the emotional complexity and uncertainty surrounding decisions about risk-reducing surgery, national guidelines such as those from NICE (National Institute for Health and Care Excellence) recommend that these decisions be supported in specialist multi-disciplinary clinics [54, 55]. Such settings provide time and space to weigh risks and benefits, ideally with access to specialists including surgeons, genetic counsellors, clinical geneticists, nurses and psychologists. Although this model offers comprehensive and patient-centred care, multidisciplinary clinics can be difficult to establish and sustain, particularly where workforce, time and financial resources are limited.

### Drivers for change

While counselling models were becoming more psychologically informed, parallel advances in genomic technology and oncology were reshaping when, how and by whom genetic testing was delivered [1, 2, 56]. The emergence of next-generation sequencing at substantially lower cost transformed testing strategies from single-gene or small-panel approaches to high-throughput multigene panels and, increasingly, whole genome sequencing [57, 58]. At the same time, the growing therapeutic relevance of genomic findings, including the use of PARP inhibitors for BRCA-associated tumours [21, 59] or immunotherapy for colorectal tumours with deficient mismatch repair (dMMR) [23, 60], increased demand for genetic testing at the point of diagnosis.

Universal tumour screening for dMMR in colorectal and endometrial cancers is now recommended in several countries, including England, Canada, Australia, Denmark and the Netherlands [25, 61–64]. These pathways typically include reflex testing for *MLH1* promoter hypermethylation or the *BRAF* V600E variant, followed by germline genetic testing where indicated, enabling identification of Lynch syndrome [65]. Paired somatic and germline testing may also identify other hereditary cancer predispositions while simultaneously guiding treatment for the presenting malignancy [17, 66–68]. In some cases, this also reveals previously unrecognised increased lifetime risks of other primary cancers unrelated to the index diagnosis [69].

Consequently, genomic testing in oncology has expanded the number of individuals and families identified as being at increased cancer risk, creating opportunities for enhanced surveillance and primary prevention before cancer develops. Studies suggest that these pathways may be cost-effective, and in some settings cost-saving, particularly when testing pathways are streamlined and familial benefits are considered [70–73]. In parallel, population-based screening pilots and research studies have been initiated in some settings to identify individuals without a known family history who wish to engage in proactive health management [74]. These initiatives increase the complexity of counselling, since cancer risk penetrance may be lower and less predictable in these populations, requiring more personalised assessment and management [75].

### Mainstreaming genetic testing

Against this backdrop, cancer genetics has been at the forefront of efforts to mainstream testing within routine clinical care [16, 27, 76–78]. Although genomics training is increasingly incorporated into curricula, many non-genetics specialists report limited confidence in discussing complex and rapidly evolving genomic information with patients [28].

Consequently, genetics services continue to provide advice and education. National education programmes have been developed to improve genomic literacy and confidence among mainstream clinicians, supporting integration of genomic medicine into everyday practice [79, 80]. In parallel, innovations such as integration with electronic health records and digital testing platforms have emerged, although further research is needed to evaluate their effectiveness and implementation at scale across diverse populations [81–83].

Clear, well-defined pathways are needed to identify eligible patients, streamline pre-test consent, deliver high-throughput and cost-effective laboratory testing and ensure accurate interpretation and timely communication of results [84]. Joined-up, patient-centred communication across multidisciplinary teams remains challenging, but is essential to realise the potential benefits of genomically informed cancer care [85]. In England, these priorities are reflected in the National Cancer Plan (2026), which positions genomics as a core component of modern cancer care and emphasises personalised and equitable approaches to improving outcomes [86].

### The English national lynch syndrome transformation project: a paradigm for genomic integration

The English National Lynch Syndrome Transformation project exemplifies how the principles of mainstreaming genetic testing, workforce education and patient-centred pathway design can be operationalised at national scale [87]. The programme demonstrates how genomics can be embedded within routine cancer care while maintaining specialist oversight and long-term follow-up for individuals and families with inherited cancer risk [88].

In 2022, a nationally coordinated implementation programme was launched, with genomic champions embedded within every colorectal and endometrial cancer multidisciplinary team (MDT). These champions were designated individuals who promoted pathway implementation, supported colleagues with genomics-related issues and acted as local points of contact with specialist genetics services. This model supported mainstreaming by placing responsibility for initiating and tracking testing within cancer teams, while maintaining support from genetics specialists. The genomics champions played an important role in clinician education, pathway adherence and accountability, helping to address gaps in genomic literacy and confidence among non-genetics specialists [88].

Patients with dMMR tumours were then supported to proceed to germline testing where indicated, either through referral to genetics services or through mainstream testing after appropriate consent discussions within cancer services [87]. Individuals identified with clinically actionable (likely pathogenic or pathogenic) variants in MMR genes were referred to clinical genetic services for counselling and long-term management, including tailored surveillance, consideration of aspirin as a precision prevention strategy and discussion of risk-reducing surgery where appropriate. Predictive testing for at-risk relatives was coordinated through clinical genetic services, supporting families to navigate the lifelong implications of a Lynch syndrome diagnosis.

To address regional variation in access to colonoscopy, all individuals with Lynch syndrome were entered into a national, quality-assured bowel screening programme with recall systems designed to support surveillance at appropriate intervals. A national Lynch Syndrome Register was developed by the English National Disease Registration Service (NDRS) to provide a single point of referral into the screening programme and a comprehensive list of individuals with Lynch syndrome in England [89, 90]. Retrospective and prospective case ascertainment through regional genetics services (from July 2023) identified 9030 existing carriers to populate the register [91].

The design and delivery of the programme were informed by patient and public involvement (PPI), including advocacy organisations such as Lynch Syndrome UK and lived-experience input. These contributors highlighted the importance of clear clinical ownership, timely communication and reliable long-term follow-up. Their priorities were reflected in the development of standardised pathways and national recall systems, which reduced the burden on patients to coordinate their own surveillance.

Building on this infrastructure, the Lynch Syndrome Portal has subsequently been adopted as the model for the National Inherited Cancer Predisposition Register, also hosted by the NDRS under the same legal framework as the National Cancer Registration Service [91]. This expanded register aims to identify all individuals with pathogenic or likely pathogenic variants across a broader range of cancer predisposition genes, extending the principles established through the Lynch syndrome programme.

#### The evolving role of genetics services in mainstreamed cancer care

Mainstreaming genetic testing has reshaped the role of specialised genetics services and demanded substantial service transformation [16, 28]. Follow-up genetic counselling may include post-test referrals for surveillance and risk-reducing interventions, psychosocial support, research participation and links to peer support organisations and charities [92]. Cascade predictive genetic testing for at-risk relatives, most of whom are unaffected by cancer, remains a core responsibility of genetics services and is central to the preventative potential of genomic medicine [93]. Demand for genetic counselling services remains high, reflecting both increased case ascertainment and the expanding scope of genomic medicine [16].

To support service sustainability, new workforce roles have emerged. These have varied internationally, including genomics associates, genomics practitioners and genetic counselling extenders [13, 94, 95]. Genomics associates typically support administrative and pathway coordination functions. Genomics practitioners have additional training in genomic medicine and may contribute to case management, tracking results and service delivery under supervision. Genetic counselling extenders are nurses trained to provide cancer risk assessment and genetic testing after triage by a genetic counsellor [96]. These roles allow genetics specialists to focus on clinical interpretation, genetic counselling and shared decision-making [28]. They may also provide a route into the profession, with support workers gaining experience before progressing to formal training programmes to become clinicians [2].

Importantly, the traditional genetic counselling model, including comprehensive pre-test support, remains essential for selected individuals diagnosed with cancer [1]. This includes patients with additional communication or support needs, such as language interpretation, learning difficulties or mental health challenges, distress or difficulty processing complex genomic information [36]. Clinical judgement is therefore essential to triage patients appropriately for early involvement of genetics or psychological services, ensuring care remains responsive and patient-centred [47, 97].

The increasing use of digital infrastructure, including online consent platforms, patient information leaflets, videos and decision support interventions, has further supported the development of streamlined and proportionate pathways [84, 98, 99]. Together, these developments show how genetics services have adapted to the provision of mainstreamed testing while retaining their essential role in expert interpretation, psychosocial support and longitudinal family-centred care.

### Personalised risk assessment in contemporary genetic counselling

Personalised risk assessment is a core component of genetic counselling for hereditary cancer, reflecting advances in genomic testing, risk penetrance estimates and precision prevention [100, 101]. Risk is no longer understood solely by a single high-penetrance gene variant, but increasingly as probabilistic, evolving and shaped by interacting genetic and non-genetic factors across the life course [75]. Interpretation of genetic variants is evolving [102]. Variants of uncertain significance may be reclassified over time, raising responsibility for recontact about important updates [103]. Cancer risks associated with known pathogenic variants may also become more precisely defined as larger datasets emerge [104]. As a result, genetic counselling increasingly involves helping individuals understand that risk information is provisional and may change, rather than presenting fixed estimates as definitive [105, 106].

Alongside technical developments, the psychological and ethical dimensions of risk assessment have become more visible. Probabilistic estimates can be difficult to interpret and may generate anxiety, decisional conflict or a sense of ongoing vulnerability [107]. This is especially relevant when management choices can have irreversible consequences. Effective counselling therefore requires longitudinal support to help revisit decisions over time and integrate genetic risk into a broader understanding of health and identity [108, 109].

Attention to health disparities is essential as genomic medicine becomes more sophisticated. Many genomic datasets are derived predominantly from populations of European ancestry, limiting generalisability [110]. Genetic counselling should therefore be delivered in a way that is culturally responsive, recognising, respecting and adapting to individuals’ beliefs, language needs and social circumstances to support informed choice rather than prescriptive decision-making [111].

Genetic counselling provides an opportunity to consider a holistic approach to health, during which individuals may be receptive to discussions about the interaction between inherited cancer risk and modifiable diet, lifestyle, environmental and other non-genetic factors [112]. Decisions about testing, surveillance and risk-reducing interventions often intersect with broader life priorities and motivations for behaviour change [113]. Genetic counselling increasingly involves supporting patients when test results are unexpected or evidence is incomplete [114]. More research is needed to understand how a broader counselling approach might support healthier behaviour change, for example adopting a high fibre diet to reduce colorectal cancer risk, but the magnitude of such benefits should be framed cautiously in the context of a high-risk inherited cancer predisposition like Lynch syndrome [115, 116].

**Fig. 1:**
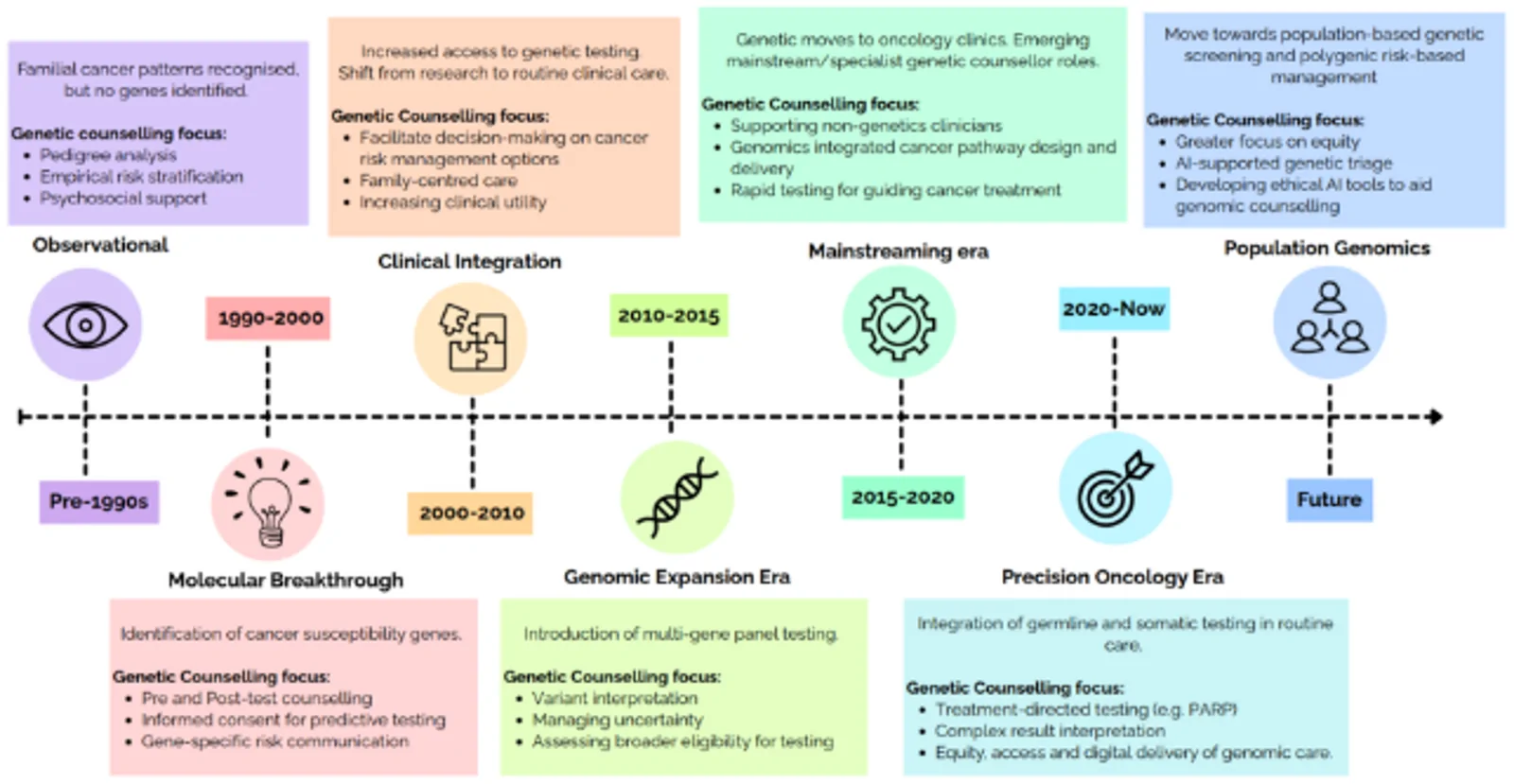
Timeline for the evolution of genetic counselling for inherited cancer predisposition

In the current landscape, the core principles of genetic counselling—shared decision-making, values clarification and support for autonomy—remain central [2, 117]. Genetic counselling aims to support individuals to make informed decisions that align with their tolerance for risk and uncertainty, while maintaining trust in both current knowledge and the evolving nature of genomic science [1]. This personalised approach remains essential to sustainable and acceptable hereditary cancer care in an era of increasingly complex risk information. Figure 1 summarises the parallel evolution of genetic counselling practice, genomic technologies and oncology integration.

### Patient experience and outcomes

Patients are the experts in their own lives [118]. Listening to the patient voice is essential to shaping genetic counselling services that reflect lived experience as well as scientific evidence and clinical theory [1]. Decision-making about inherited cancer risk is often emotionally demanding and influenced by social context, co-existing health conditions, prior experiences of cancer and known or hidden barriers to accessing care [47]. There may be challenges related to family communication, data privacy and relational responsibility [119, 120]. Patients may need support when sharing genetic information with relatives, often benefitting from specialist genetics input [121–123]. These issues reinforce the importance of longitudinal genetic counselling relationships, rather than a single consultation model.

Patient reported outcomes (PRO) provide an important means of capturing the psychosocial dimensions of genetic counselling, including knowledge, self-perceived cancer risk, anxiety, distress and satisfaction with testing and care [124]. To date, this literature has focused predominantly on hereditary breast and ovarian cancer, with fewer studies in Lynch syndrome and other predispositions [125]. Evidence from underserved and marginalised populations is also limited, which remains an important gap. Although multiple PRO measures exist, the lack of a standardised core set limits comparison across conditions, populations and healthcare settings which could help to support service evaluation [126].

The Genetic Counselling Outcomes Scale (GCOS-24) is a validated PRO measure centred on empowerment, a core aim of genetic counselling [127]. Its development was informed by qualitative research exploring what matters most to patients accessing genetics services [127]. While originally designed for broad use across genetic counselling indications, shorter versions may be more feasible for in mainstreamed pathways [128]. Greater harmonisation of PRO measures, drawing on established oncology approaches [129], could strengthen evaluation of services and support continuous improvement.

### Patient and public involvement (PPI)

PPI is integral to understanding and improving the experience of genetic counselling and inherited cancer care. This review includes two patient representatives with lived experience of Lynch syndrome (JY, BB) as co-authors who provided narrative accounts of experiences with genetic counselling for Lynch syndrome. This did not constitute qualitative research, but rather it was agreed as a flexible PPI approach for original contribution to the drafting of the manuscript. The full narratives are in Supplementary File 1. Table 1 includes key points from the narratives that highlight the main themes in this review, including the impact of mainstreaming, communication and coordinated care. Taken together, these narratives show that genetic counselling is most helpful when it combines technical explanation with emotional support, practical coordination, and family-centred communication. They also reinforce the continuing need for specialist genetics input within mainstreamed pathways, particularly where patients require ongoing support across the life course.

**Table 1:** Key points from patient co-author narratives, highlighted the themes explored in this review, including mainstreaming, communication and coordinated care

Key themes and sub-themes	Points raised from JY’s story	Points raised from BB’s story
Mainstreaming genetic testing: benefits and areas for development Knowledgeable non-genetics specialists (education) Time pressures (protected time for mainstream clinicians to deliver genetic testing) Continuity of care (named clinician with link to genetics) Quicker results	When I was diagnosed with colorectal cancer, I did actively choose to have the same consultant as my dad and ultimately the same oncologist. This meant that I was tested and diagnosed quite quickly with Lynch Syndrome Variant MSH6. This for me was incredibly important as I had family of my own that I was keen to protect whenever and however I could It isn’t aways their fault, they are so pushed for time and so they don’t have the time or compassion to talk over the impact of genetic testing and the involvement of other family members	In terms of patient experience, I think having an decreased turn- around time from getting tested, to getting the results would help to reduce the waiting anxiety that comes with the unknown. However, I understand the resources and staffing pressures that is present in medical care so this may be just too difficult I have had a specific person who has always answered my questions and even taken calls at times of need. I find this part of the genetic counselling very reassuring and I would definitely rather have this person than not
Communication: Access to genetic counselling (still needed despite mainstream implementation) Familial risk (specialist area of genetic counselling)	In my opinion, I believe the role of the genetic counsellor to be imperative, and I honestly can imagine my journey being harder without their input It has not been uncommon to speak to friends from social media groups to find that the time frame from being tested to the results being disseminated are very long and this can be such an anxious time for some There was also the realisation that future grandchildren would be at risk too. I think for me having a cancer diagnosis that was easy but for other family members, it was a lot to take on board	I met with a genetic counsellor who explained more about the syndrome, the testing process and future screening. This was very useful for me as on occasion, I would find myself going down a rabbit hole of Google when actually less information that is more evidenced based is much better for my mental health when thinking about Lynch Syndrome Since being diagnosed I have had two children who may or may not have the mismatched gene which I hope through time, research and trials will be able to minimise risks massively in their lives if they are positive
Coordinated care: Specialist genetic services provide a central, ongoing coordination role Peer support Patient and public involvement (experts in lived experience)	I do think that localised support groups would be hugely helpful, both in a way of supporting each other but also for spreading awareness or even raising money as all these things make you feel so much more empowered The fact that patients views and opinions are found to be so helpful which will end up helping other patients and carers in the future is hugely rewarding. It’s a way to give back and to help others in the future and I think as patients this is what we all want	Having an itemised list of what is recommended, at what intervals and how to book them would be really helpful I do think however that not all GPs are aware of what is available. That was certainly my experience when first diagnosed This obviously makes having a consistent contact with a genetic counsellor even more important as they know your history without having the re-explain each time Researchers know the science, but as patients, I feel we know the day-to-day reality. Hopefully we can help to ensure resources are not just factually correct, but also supportive for those who perhaps are in shock
Service development priorities: scalable solutions Increased demand for testing and downstream care Current advice reflects evolving evidence base (need to update regularly)	There has to be an increase in the amount of medically trained personnel and in particular genetic counsellors which would increase testing further but also allow for further sample analysis and therefore further data which will be huge for future generations	I think the process of genetic counselling has become more efficient and the process allows for more people to benefit over their lifetime For me, knowing the science helps to feel slightly more in control With the written information, particularly in the early days (it has got much better more recently), it was difficult on occasion to follow the medical jargon and break down the different variants of Lynch and the associated risks with each one. This would’ve been useful to know from the off

Full narratives are found in Supplementary File 1

PPI in this review is reported in accordance with the GRIPP2-SF (Guidance for Reporting Involvement of Patients and the Public—Short Form) framework [130], which addresses the aims, methods, results, impact and reflections (Supplementary File 2). Integrating lived experience alongside clinical and academic perspectives strengthens interpretation of the evidence base and helps ensure that the review remains grounded in the realities of navigating life with Lynch syndrome.

### Communicating on and off social media

Individuals with hereditary cancer predisposition have changing information needs across their health journey, shaped by family history and personal experience [131–134]. While healthcare professionals continue to play a key role to meet these needs [135], social media have emerged as an important space for peer support, experiential knowledge and self-advocacy [136]. Connecting with others who share similar risks and decision-making challenges can foster understanding, reduce isolation and help individuals navigate a range of topics including surveillance, risk-reducing strategies and family communication [137], further broadening knowledge beyond what is gained from medical practitioners.

Despite social media encouraging connection in many ways, it is not universally beneficial. Individuals may struggle to identify with dominant narratives, particularly where certain groups are underrepresented, such as non-white and non-binary individuals [138]. Social media can also expose individuals, particularly young people, or those newly diagnosed, to misinformation, overwhelming advice or unsolicited content. Thus, while online spaces can complement clinical care, they may also create new challenges in how hereditary cancer care experiences are represented and encountered.

## Conclusion

The evolution of genetic counselling for hereditary cancer predisposition reflects broader shifts in genomic medicine, precision oncology and patient-centred care. Using Lynch syndrome as a paradigmatic example, this review illustrates how mainstreamed genetic testing has improved access to diagnosis, treatment, prevention and family-based risk management, while reshaping the role of specialist genetics services. As testing becomes increasingly embedded within oncology practice, high-quality quality care depends not only on access to testing but also on personalised counselling, clear pathways and ongoing support as patients and families navigate complex and sometimes uncertain information.

The experiences shared by patient co-authors highlight the importance of timely communication, continuity of care and practical support for surveillance, decision-making and cascade testing. They also emphasise that individuals seek information and support across multiple settings, including healthcare services, patient communities and social media. Genetic counselling therefore remains most valuable when it addresses both the technical and psychosocial dimensions of hereditary cancer risk and adapts to individuals’ changing needs over time.

Lynch syndrome illustrates how genomic medicine can be successfully integrated at scale into routine care when clinical pathways, workforce education, specialist expertise and patient-centred communication are aligned. The principles outlined in this review, including equitable access, longitudinal family-centred care and support for informed, shared decision-making, have relevance beyond Lynch syndrome and will remain central to the continued evolution of genetic counselling in cancer care.

## Supplementary Information

Below is the link to the electronic supplementary material.Supplementary Material 1.

## Data Availability

No datasets were generated or analysed for this review.
