# Supplementary material for: The evolution of hereditary cancer genetic counselling: mainstreaming, service redesign and patient experience in Lynch syndrome
Source: Fam Cancer. 2026 Aug 29;25(3):84. doi: 10.1007/s10689-026-00597-6 (PMC13526072; doi:10.1007/s10689-026-00597-6)
Supplement: Supplementary file 1 [file 10689_2026_597_MOESM1_ESM.docx]

**Supplementary File 1. Perspectives from lived experience with genetic counselling for Lynch syndrome: narratives from patient co-authors**

| **Question** | **JY’s story** | **BB’s story** |
| --- | --- | --- |
| **What was it was like to have genetic counselling?** | When I was diagnosed with colorectal cancer at the age of 44 I was already somewhat aware of a family history of cancer having had both an interest in family history and also having recently helped care for my own dad through his cancer journey and so by the time I first saw my surgeon in clinic I had researched my dad's consultant and knew that he had an interest in family history of disease and so I did actively choose to have the same consultant as my dad and ultimately the same oncologist. This meant that I was tested and diagnosed quite quickly with Lynch Syndrome Variant MSH6. This for me was incredibly important as I had family of my own that I was keen to protect whenever and however I could.  The genetic counsellor explained in depth what a change in a gene meant and the risks it posed. It was quite evident that there was a strong history in our family. She also discussed screening and the possibility of preventative surgery. I talked to her about my worry for my sons and my extended family and agreed that I would distribute letters to my family members. This was easy to an extent initially but got a little complicated as there was a huge family rift and my sons were in their early twenties and were very resistant to begin testing and they held off from doing this for almost five years.  I found the information from my genetic counsellor extremely helpful at the time, and it was key to beginning a journey of discovery. Sadly, other family members were not as enthusiastic although most of my family did get tested too, there were some mixed emotions for them such as guilt for passing it on or guilt when a family member tested negative and others positive.  There was also the realisation that future grandchildren would be at risk too. I think for me having a cancer diagnosis that was easy but for other family members, it was a lot to take on board. | I was diagnosed with Lynch Syndrome (MSH2) on Christmas Eve at the age of 30. My mum disclosed she was Lynch positive and that I may have a 50/50 chance of having it. At the time, I hadn’t heard of it at all and had not even thought much about cancer until that point due to having a healthy lifestyle and perhaps a little ignorance.  Before my Lynch positive results came through, I met with a genetic counsellor who explained more about the syndrome, the testing process and future screening. This was very useful for me as on occasion, I would find myself going down a rabbit hole of google when actually less information that is more evidenced based is much better for my mental health when thinking about Lynch Syndrome.  Once confirmed as positive, my counsellor quickly arranged a colonoscopy at my local hospital which thankfully came back clear. I think having a counsellor at this stage was hugely beneficial as I felt I had a direct contact to not only answer any questions but also arrange any screening. I have had a specific person who has always answered my questions and even taken calls at times of need. I find this part of the genetic counselling very reassuring and I would definitely rather have this person than not.  Since being diagnosed I have had two children who may or may not have the mismatched gene which I hope through time, research and trials will be able to minimise risks massively in their lives if they are positive. |
| **How has genetic counselling changed over time?** | It has not been uncommon to speak to friends from social media groups to find that the time frame from being tested to the results being disseminated are very long and this can be such an anxious time for some. For my family members, I think we were tested reasonably quickly, for myself those results came quickly as they did have my tumour to test and I believe this made it an easier task. My siblings’ results took around 6 weeks. One massive change I have seen is that the knowledge of Lynch Syndrome is becoming a talking point now and medical professionals are far more aware than they used to be. | I believe the awareness of Lynch Syndrome has been much more apparent over the last 10 years. I know there is a slight bias because I am now drawn towards more ‘colon cancer’ type articles and actively search research trials, however I think it has a higher profile particularly with more celebrities getting the cancer, so just more prominent in the news.  I have younger siblings; one who went through the testing, diagnosing and is positive, now actively going through the screening programme. The whole process for them happened within a couple of months, so very quickly.  I think the process of genetic counselling has become more efficient and the process allows for more people to benefit over their lifetime. |
| **What has been the impact of mainstream genetic testing in routine cancer care?** | In my opinion, I believe the role of the genetic counsellor to be imperative, and I honestly can imagine my journey being harder without their input. Consultants and nursing staff are surprisingly impartial to passing on information or empathising in a clinic situation (my opinion). It isn’t aways their fault, they are so pushed for time and so they don’t have the time or compassion to talk over the impact of genetic testing and the involvement of other family members. I really believe this can only be done more thoroughly in an appointment with a genetic counsellor. This is their speciality and so they have more knowledge but are also so much more empathetic in clinic. | I am currently in a very fortunate position where I am approaching 40 and am so far cancer free and have had 5 clear colonoscopies. So, in ‘routine cancer care’ I find it difficult to comment.  I believe that my families Lynch Syndrome was picked up in tumour testing in my grandmother’s cancer many years ago. This prompted my mother and her siblings to get tested for Lynch and then subsequently any children. So, this experience starting with my grandmother’s testing meant that as a family we can minimise our risks through screening programmes.  I do think however that not all GPs are aware of what is available. That was certainly my experience when first diagnosed. |
| **Is there any information or support that is missing or could be improved for patients?** | I don’t remember at my own genetics appointment, although I had a positive experience, having been told that I could make another appointment at another time if I needed to chat.  I do think that localised support groups would be hugely helpful, both in a way of supporting each other but also for spreading awareness or even raising money as all these things make you feel so much more empowered. I have found a huge amount of my information more recently through the Lynch Syndrome UK Facebook group which is up to date and people help people with their experiences and stories. | When I was first diagnosed the main information pushed was the colonoscopy and the *H. Pylori* test. It has only been more recently that I have understood the need to get skin checks as part of the screening programme. Having an itemised list of what is recommended, at what intervals and how to book them would be really helpful.  I also have a very busy job and a hectic family life – this has made attending day time appointments more challenging. |
| **How has more scientific knowledge influenced patient care?** | The advances in the knowledge of genetic variants have grown exponentially. This has meant a huge uptake in patients being tested and the same growth in preventative surgery and also regular screening which in the short term must be a huge burden on the health services we have but it also will highlight the lack of fully trained professionals. Further complications to this are the advances we have seen for treatments in hereditary cancerThe medical field is constantly metamorphosising and as new variants are discovered new treatments are also discovered and I’m sure this will always be the case. | More scientific research has definitely influenced the frequency and ease of colonoscopies every two years. One of the more recent positive additions has been the screening programmes pro-active approach to booking this all in. My last three colonoscopies have been instigated by the hospital which first starts with a telephone call and then very quickly followed by the procedure – typically all done within a couple of weeks.  When first diagnosed, I enrolled in the CaPP3 aspirin study and was sent aspirin as part of the trial. I understand that once completed, Capp3 concluded that low dose aspirin can help in reducing the risk of polyps/bowel cancer. This is something I continued with for a couple of years.  For me, knowing the science helps to feel slightly more in control. |
| **How hard is it for people to get the right information for them in a way that is easy to understand?** | I do think it can be hard to get the right information; this is getting better with further knowledge but there is still a good way to go. Some people with one variant can have a huge difference in familial risk and seemingly add questions to the information we currently receive. For example, one person with variant MSH2 may never develop cancer, and others will develop multiple cancers.  There are also the new developments in preventative medication such as aspirin which have proved helpful in reducing the risk of gastrointestinal cancers. However, some people can take aspirin, and some cannot. The aspirin may work for some and not for others and this can only be quantified after a lengthy time. | I think when you are in person with your genetic counsellor, it is far easier to understand the information as it can be explained there and then. With the written information, particularly in the early days (it has got much better more recently), it was difficult on occasion to follow the medical jargon and break down the different variants of Lynch and the associated risks with each one. This would’ve been useful to know from the off. |
| **How could patient experience be made better?** | I do believe things are in general, heading in the right direction with the increase of those being diagnosed. However, I do also believe that strength is in numbers and so there has to be an increase in the amount of medically trained personnel and in particular genetic counsellors which would increase testing further but also allow for further sample analysis and therefore further data which will be huge for future generations. This would also give a really positive message to those people who are newly diagnosed and at the beginning for their journey. | I think that this probably starts with the GP. I am not a regular visitor to ‘the doctor’, no more than once or twice per year, however it seems to always be someone different. This obviously makes having a consistent contact with a genetic counsellor even more important as they know your history without having the re-explain each time.  In terms of patient experience, I think having an decreased turn around time from getting tested, to getting the results would help to reduce the waiting anxiety that comes with the unknown. However I understand the resources and staffing pressures that is present in medical care so this may be just too difficult. |
| **How important is patient involvement in research and codesign of resources?** | I have found helping with research projects in this field to be so therapeutic. The whole process of helping with online meetings, face to face meetings with groups of patients and professionals and the eventual outcomes of new documents to be distributed to be so rewarding and have met and made lifelong friends with so many wonderful people on this path. The fact that patients views and opinions are found to be so helpful which will end up helping other patients and carers in the future is hugely rewarding. It’s a way to give back and to help others in the future and I think as patients this is what we all want | From a patients perspective, I think having involvement from real people is vital. I have taken part in a couple of research tasks and have not only found it helps me to feel more in control of the syndrome but also because I am ‘the average person’ it may help others who are newly diagnosed.  Researchers know the science, but as patients, I feel we know the day to day reality. Hopefully we can help to ensure resources are not just factually correct, but also supportive for those who perhaps are in shock. |

**Supplementary File 2. Reporting of PPI in the review, guided by GRIPP2-SF (G**uidance for **R**eporting **I**nvolvement of **P**atients and the **P**ublic – **S**hort **F**orm)

| **Aim** | To incorporate lived experience of Lynch syndrome into the planning, interpretation and reporting of this review, with a particular focus on experiences of genetic counselling and communication of inherited cancer risk. |
| --- | --- |
| **Methods** | Two patient representatives with lived experience of Lynch syndrome (JY, BB) were included as co‑authors. They contributed to the planning, writing and approval of the final manuscript and provided narrative accounts informed by their personal experiences and interactions with family members and peers through Lynch Syndrome UK and other patient networks. |
| **Results** | Patient narratives highlighted key themes related to genetic counselling for Lynch syndrome, which are presented in Table 1. These contributions provided patient‑centred insights into counselling experiences, communication challenges and psychosocial considerations. |
| **Impact** | PPI informed the framing and interpretation of the review, strengthened emphasis on patient‑centred communication and counselling, and shaped recommendations related to care pathways and support needs for individuals and families with Lynch syndrome. |
| **Reflections** | Inclusion of patient representatives as authors enabled meaningful integration of lived experience. However, only two representatives could be included due to time and funding limitations. Perspectives may particularly reflect individuals engaged with patient advocacy networks. Broader representation of diverse patient experiences should be considered in future work. This is likely to require dedicated investment both in terms of time, funding and building trusted relationships in communities. |
